# Supplementary figures and images for: Poa annua: An annual species?
Source: PLoS One. 2022 Sep 9;17(9):e0274404. doi: 10.1371/journal.pone.0274404 (PMC9462799; doi:10.1371/journal.pone.0274404)

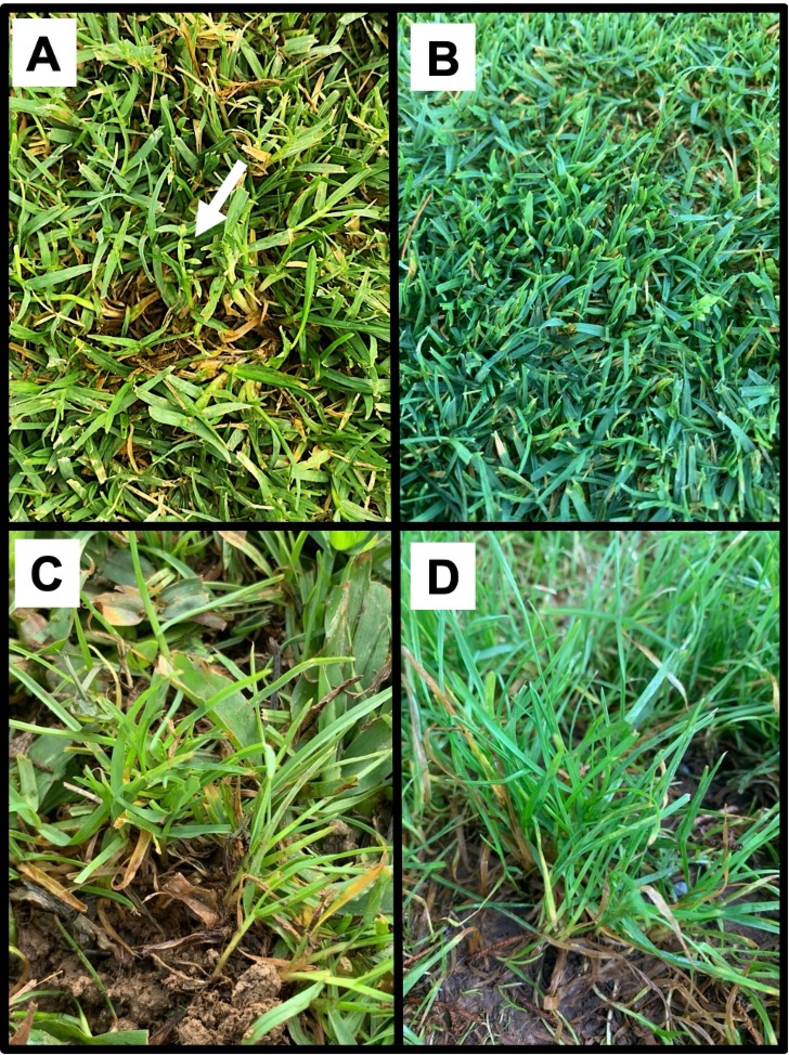

Supplement: S1 Fig — Images taken on 4 August 2020 where A = a fine-texture, laterally growing plant with inflorescence in micro-environment two; B = a fine-texture, laterally growing plant in micro-environment six; C = an upright growing, coarse textured plant in micro-environment seven; and D = an upright growing, coarse textured plant in micro-environment nine. (TIF) [file pone.0274404.s001.tif]
