# Supplementary material for: Poa annua: An annual species?
Source: PLoS One. 2022 Sep 9;17(9):e0274404. doi: 10.1371/journal.pone.0274404 (PMC9462799; doi:10.1371/journal.pone.0274404)
Supplement: S1 Table — (DOCX) [file pone.0274404.s002.docx]

S1 Table

| **Experiment** | **Dates** | **Location** |
| --- | --- | --- |
| Life cycle observation | 11 May to 27 October 2020 | Field |
| Soil temperature evaluation | 24 February to 14 April 2021 | Greenhouse |
| Soil water evaluation | 21 April to 9 June 2021 | Greenhouse |
| Disease susceptibility evaluation | 26 May to 13 October 2021 | Field |
