# Supplementary material for: Poa annua: An annual species?
Source: PLoS One. 2022 Sep 9;17(9):e0274404. doi: 10.1371/journal.pone.0274404 (PMC9462799; doi:10.1371/journal.pone.0274404)
Supplement: S2 Table — (DOCX) [file pone.0274404.s003.docx]

S2 Table

| Application date | Fungicide trade name | Producing company  (brand, city, state) | Active ingredient rate (ha^-1^) | FRAC^†^ mode of action group number |
| --- | --- | --- | --- | --- |
| 27 May | Heritage Action  Posterity | Syngenta, Research Triangle Park, NC  Syngenta, Research Triangle Park, NC | 0.3 kg azoxystrobin  0.05 kg pydifluometofen | 11  7 |
| 10 June | Maxtima  Chipco Signature | BASF Corporation, Research Triangle Park, NC  Bayer Environmental Science, Cary, NC | 0.76 kg mefentrifluconazole  9.8 kg aluminum tris | 3  2 |
| 24 June | Daconil Ultrex  Segway | Syngenta, Research Triangle Park, NC  PBI Gordon Corporation, Shawnee, KS | 9.2 kg clorothalonil  0.72 kg cyazofamid | 5  21 |
| 8 July | Insignia  Chipco Signature | BASF Corporation, Research Triangle Park, NC  Bayer Environmental Science, Cary, NC | 0.71 kg pyraclostrobin  9.8 kg aluminum tris | 11  2 |
| 22 July | Ascernity  Segway | Syngenta, Research Triangle Park, NC  PBI Gordon Corporation, Shawnee, KS | 0.07 kg benzovindiflupyr + 0.25 kg difenoconazole  0.72 kg cyazofamid | 7  21 |
| 4 Aug | Chipco Signature  Rayora | Bayer Environmental Science, Cary, NC  FMC, Philadelphia, PA | 9.8 kg aluminum tris  0.6 kg flutriafol | 2  3 |
| 18 Aug | Heritage Action  Daconil Ultrex  Segway | Syngenta, Research Triangle, NC  Syngenta, Research Triangle Park, NC  PBI Gordon Corporation, Shawnee, KS | 0.3 kg azoxystrobin  9.2 kg clorothalonil  0.72 kg cyazofamid | 11  5  21 |
| 2 Sep | Maxtima  Chipco Signature | BASF Corporation, Research Triangle Park, NC  Bayer Environmental Science, Cary, NC | 0.76 kg mefentrifluconazole  9.8 kg aluminum tris | 3  2 |
| 16 Sep | Insignia  Posterity | BASF Corporation, Research Triangle Park, NC  Syngenta, Research Triangle, NC | 0.71 kg pyraclostrobin  0.05 kg pydifluometofen | 11  7 |
| 30 Sep | Maxtima  Segway | BASF Corporation, Research Triangle Park, NC  PBI Gordon Corporation, Shawnee, KS | 0.76 kg mefentrifluconazole  0.72 kg cyazofamid | 3  21 |
| 13 Oct | Daconil Ultrex  Heritage Action | Syngenta, Research Triangle Park, NC  Syngenta, Research Triangle, NC | 9.2 kg clorothalonil  0.3 kg azoxystrobin | 5  11 |
