# Supplementary material for: Poa annua: An annual species?
Source: PLoS One. 2022 Sep 9;17(9):e0274404. doi: 10.1371/journal.pone.0274404 (PMC9462799; doi:10.1371/journal.pone.0274404)

PLOS ONE

Poa annua: An annual species?

--Manuscript Draft--

**Manuscript Number:**	PONE-D-22-14930
**Article Type:**	Research Article
**Full Title:**	Poa annua: An annual species?
**Short Title:**	Poa annua life cycle
**Corresponding Author:**	James T Brosnan, Ph.D.<br>University of Tennessee<br>Knoxville, TN UNITED STATES
**Keywords:**	Poa annua; annual bluegrass; turfgrass; life cycle; management
**Abstract:**	As the Latin name annua implies, the species Poa annua L. is thought to have an annual life cycle. Yet, there are many reports in literature of P. annua persisting as a perennial. Considering that P. annua senescence patterns do not align with other true annual species, we hypothesized that P. annua is similar to other perennial, C3 turfgrass species that are subject to a confluence of environmental factors that can cause mortality. Four experiments were conducted in Knoxville, TN with the objective of determining environmental factors lethal to P. annua. A field monitoring study assessed 100 P. annua plants across ten grassland micro-environments from May to October 2020. Forty plants survived the summer and confirmed the existence of perennial P. annua ecotypes. Analysis of environmental factors at the time of plant death indicated soil moisture, soil temperature, and pathogenic infection were associated with mortality. A series of glasshouse or field experiments were conducted to investigate the effects of each factor on P. annua mortality. Soil moisture and soil temperature were not lethal to P. annua in the glasshouse, except under extreme conditions not typical in the field. A field study assessed mortality of plants from pathogenic infection and indicated that P. annua plants treated with fungicide throughout the summer survived year-round, whereas plants not receiving fungicide applications senesced. These findings support our hypothesis that P. annua is of a perennial life cycle, which can be influenced by environmental conditions. We suggest that the name P. annua is likely a misnomer based on its modern interpretation.
**Order of Authors:**	Devon E Carroll, Ph.D.
	Brandon J. Horvath
	Michael Prorock
	Robert N. Trigiano
	Avat Shekoofa
	Thomas C. Mueller
	James T. Brosnan
**Additional Information:**	
**Question**	**Response**
**Financial Disclosure**<br><br>Enter a financial disclosure statement that describes the sources of funding for the work included in this submission. Review the submission guidelines for detailed requirements. View published research articles from *PLOS ONE* for specific examples.	The author(s) received no specific funding for this work.

Powered by Editorial Manager® and ProduXion Manager® from Aries Systems Corporation

This statement is required for submission and **will appear in the published article** if the submission is accepted. Please make sure it is accurate.<br><br>**Unfunded studies**<br>Enter: *The author(s) received no specific funding for this work.*<br><br>**Funded studies**<br>Enter a statement with the following details:<br>• Initials of the authors who received each award<br>• Grant numbers awarded to each author<br>• The full name of each funder<br>• URL of each funder website<br>• Did the sponsors or funders play any role in the study design, data collection and analysis, decision to publish, or preparation of the manuscript?<br>• **NO** - Include this sentence at the end of your statement: *The funders had no role in study design, data collection and analysis, decision to publish, or preparation of the manuscript.*<br>• **YES** - Specify the role(s) played.<br><br>* typeset	
**Competing Interests**<br><br>Use the instructions below to enter a competing interest statement for this submission. On behalf of all authors, disclose any competing interests that could be perceived to bias this work—acknowledging all financial support and any other relevant financial or non-financial competing interests.<br><br>This statement is required for submission and **will appear in the published article** if the submission is accepted. Please make sure it is accurate and that any funding sources listed in your Funding Information later in the submission form are also declared in your Financial Disclosure statement.<br><br>View published research articles from *PLOS ONE* for specific examples.	The authors have declared that no competing interests exist.

**NO authors have competing interests**<br><br>Enter: *The authors have declared that no competing interests exist.*<br><br>**Authors with competing interests**<br><br>Enter competing interest details beginning with this statement:<br><br>*I have read the journal's policy and the authors of this manuscript have the following competing interests: [insert competing interests here]*<br><br>* typeset	
**Ethics Statement**<br><br>Enter an ethics statement for this submission. This statement is required if the study involved:<br><br>• Human participants<br>• Human specimens or tissue<br>• Vertebrate animals or cephalopods<br>• Vertebrate embryos or tissues<br>• Field research<br><br>Write "N/A" if the submission does not require an ethics statement.<br><br>General guidance is provided below. Consult the submission guidelines for detailed instructions. **Make sure that all information entered here is included in the Methods section of the manuscript.**	Field research and sample harvesting was conducted on university property, Three Ridges Golf Course, or Oak Ridge Country Club. No permits were required for testing or plant collection. Permission for use was granted by site managers.

Powered by Editorial Manager® and ProduXion Manager® from Aries Systems Corporation

**Format for specific study types**<br><br>**Human Subject Research (involving human participants and/or tissue)**<br>• Give the name of the institutional review board or ethics committee that approved the study<br>• Include the approval number and/or a statement indicating approval of this research<br>• Indicate the form of consent obtained (written/oral) or the reason that consent was not obtained (e.g. the data were analyzed anonymously)<br><br>**Animal Research (involving vertebrate animals, embryos or tissues)**<br>• Provide the name of the Institutional Animal Care and Use Committee (IACUC) or other relevant ethics board that reviewed the study protocol, and indicate whether they approved this research or granted a formal waiver of ethical approval<br>• Include an approval number if one was obtained<br>• If the study involved *non-human primates*, add *additional details* about animal welfare and steps taken to ameliorate suffering<br>• If anesthesia, euthanasia, or any kind of animal sacrifice is part of the study, include briefly which substances and/or methods were applied<br><br>**Field Research**<br><br>Include the following details if this study involves the collection of plant, animal, or other materials from a natural setting:<br>• Field permit number<br>• Name of the institution or relevant body that granted permission	
**Data Availability**<br><br>Authors are required to make all data underlying the findings described fully available, without restriction, and from the time of publication. PLOS allows rare exceptions to address legal and ethical concerns. See the PLOS Data Policy and FAQ for detailed information.	Yes - all data are fully available without restriction

A Data Availability Statement describing where the data can be found is required at submission. Your answers to this question constitute the Data Availability Statement and **will be published in the article**, if accepted.<br><br>**Important:** Stating 'data available on request from the author' is not sufficient. If your data are only available upon request, select 'No' for the first question and explain your exceptional situation in the text box.<br><br>Do the authors confirm that all data underlying the findings described in their manuscript are fully available without restriction?	
**Describe where the data may be found in full sentences. If you are copying our sample text, replace any instances of XXX with the appropriate details.**<br><br>• If the data are **held or will be held in a public repository**, include URLs, accession numbers or DOIs. If this information will only be available after acceptance, indicate this by ticking the box below. For example: *All XXX files are available from the XXX database (accession number(s) XXX, XXX.).*<br>• If the data are all contained **within the manuscript and/or Supporting Information files**, enter the following: *All relevant data are within the manuscript and its Supporting Information files.*<br>• If neither of these applies but you are able to provide **details of access elsewhere**, with or without limitations, please do so. For example:<br><br>*Data cannot be shared publicly because of [XXX]. Data are available from the XXX Institutional Data Access / Ethics Committee (contact via XXX) for researchers who meet the criteria for access to confidential data.*<br><br>*The data underlying the results presented in the study are available from (include the name of the third party*	Data are available for experiments 1 and 4 at https://colab.research.google.com/drive/16eIDgyFMX08K2szhc_LdXmbYavf982jY?usp=sharing. Data are available for experiments 2 and 3 at https://mesurpubshare.blob.core.windows.net/public/PoaLifeCycle_PLOS_Data.xlsx.

and contact information or URL).<br>• This text is appropriate if the data are owned by a third party and authors do not have permission to share the data.<br><br>* typeset	
Additional data availability information:	

Manuscript

Click here to access/download;Manuscript;PoaLifeCycle_PLOS_V5.docx

***Poa annua*: An annual species?**

Devon E. Carroll[1*], Brandon J. Horvath[1], Michael Prorock[2], Robert N. Trigiano[3], Avat Shekoofa[1], Thomas C. Mueller[1], and James T. Brosnan[1]

[1]Department of Plant Sciences, The University of Tennessee, Knoxville, TN, USA

[2] Mesur.io, Yanceyville, NC, USA

[3] Department of Entomology and Plant Pathology, The University of Tennessee, Knoxville, TN, USA

* Corresponding author

E-mail: devcarroll24@gmail.com

Abstract

As the Latin name *annua* implies, the species *Poa annua* L. is thought to have an annual life cycle. Yet, there are many reports in literature of *P. annua* persisting as a perennial. Considering that *P. annua* senescence patterns do not align with other true annual species, we hypothesized that *P. annua* is similar to other perennial, C_3 turfgrass species that are subject to a confluence of environmental factors that can cause mortality. Four experiments were conducted in Knoxville, TN with the objective of determining environmental factors lethal to *P. annua*. A field monitoring study assessed 100 *P. annua* plants across ten grassland micro-environments from May to October 2020. Forty plants survived the summer and confirmed the existence of perennial *P. annua* ecotypes. Analysis of environmental factors at the time of plant death indicated soil moisture, soil temperature, and pathogenic infection were associated with mortality. A series of glasshouse or field experiments were conducted to investigate the effects of each factor on *P. annua* mortality. Soil moisture and soil temperature were not lethal to *P. annua* in the glasshouse, except under extreme conditions not typical in the field. A field study assessed mortality of plants from pathogenic infection and indicated that *P. annua* plants treated with fungicide throughout the summer survived year-round, whereas plants not receiving fungicide applications senesced. These findings support our hypothesis that *P. annua* is of a perennial life cycle, which can be influenced by environmental conditions. We suggest that the name *P. annua* is likely a misnomer based on its modern interpretation.

Introduction

Poa annua L. is a C_3 grass species and a common component of urban grasslands. The ubiquity of *P. annua* in terms of both distribution and abundance has been compared to that of *Homo sapiens* [1]. Although the species can be desirable, it is also recognized as the most troublesome weed of urban grasslands and second most troublesome weed of all grass crops [2].

An annual species is defined by abrupt senescence following completion of a single reproductive cycle, even if growing conditions are optimal [3]. Grass crops such as *Triticum spp*. (wheat) and *Zea mays* L. (maize) are annuals that senesce after fruiting regardless of environmental surroundings [4-5]. Despite its name meaning annual in Latin, *P. annua* does not follow the same pattern of absolute monocarpic senescence. Furthermore, reports of *P. annua* surviving as a perennial are abundant [6-30].

It is unclear why *P. annua* is considered an annual species. Carl von Linné (Carl Linnaeus) provided no criteria when naming the species; in 1753 he describes *P. annua* as, "Extended corn bluegrass with straight angles, with smoothed spikes, with a compressed, slanted top. Minor field tuberous grass. Greater red field tuberous grass. It lives on the European paths." and in 1754 states only that he identified the plant in a field in Denmark [31-32]. Perhaps the epithet *annua* is taken too literally in modern times and was originally intended to describe an annual event such as inflorescence production.

Because *P. annua* is known to copiously produce seed [13, 14, 16, 33], the *annua* epithet may be related to observations of inflorescence characteristics. While high fecundity is frequently associated with an annual life cycle [17, 34, 35] other species common to urban grasslands such as *Taraxacum offiicinale* Weber ex. Wiggers (dandelion) and *Trifolium repens* L. (white clover) are prolific in inflorescence/seed production and survive perennially [36-37].

Additionally, *P. annua* can produce seed continuously [7, 17, 19] resulting in classification as a polycarpic perennial [9, 14, 38]. By definition, a polycarpic species cannot be annual as seed is produced more than once and is not followed by plant death [3].

Could *P. annua* be a perennial species? A large-scale study of nearly 7,000 *P. annua* plants harvested across Europe (from Portugal to Finland) showed the majority of plants (5,352; 79.3%) were perennial, whereas only 1,398 (20.7%) survived less than one year [9]. This response aligns with theories of senescence and selection, which favor a perennial over annual life cycle [39-41]. For example, long-lived individuals produce more offspring over time than those with short lifespans, causing natural selection to advance toward perenniality [14, 39]. Therefore, reproductive characteristics indicate a perennial life cycle is superior for global establishment. It is more likely *P. annua* is a perennial species, given that plants have successfully colonized all seven continents across a wide range of environments [42] in potentially as few as 10,000 years [7, 43, 44].

Therefore, similar to Johnson [45], we hypothesize that *P. annua* is comparable to other perennial, C_3 urban grassland species, which are subject to a confluence of environmental factors in summer that can result in mortality. Premature senescence, particularly due to pathogenic infection, of perennial plants in summer months after seed production may give the appearance of an annual life cycle. Such an occurrence is similar in crops of *Solanum lycopersicum* L. (tomato), *S. tuberosum* L. (potato), and *Gossypium hirsutum* L. (cotton), which are perennial species that are cultivated as annuals due to premature senescence in climates with adverse temperatures [46-48].

While scientists have reported *P. annua* is highly sensitive to heat and drought stress [8, [9, 11, 13, 15, 19, 45, 49-51], the influence of these factors has often been assessed individually,

for short periods of time, and in greenhouses or growth chambers. In the field, fluctuations in temperature and moisture over time can affect disease incidence in susceptible hosts such as *P. annua* when pathogens are present.

Poa annua is susceptible to numerous diseases including dollar spot (*Clarireedia* spp.), anthracnose (*Colletotrichum cereale*), Pythium blight (*Pythium* spp.), and brown patch (*Rhizoctonia solani*) [52]. Pathology literature describes these diseases as most devastating, resulting in summer decline or plant death, when plant stress is induced via anthropogenic activities or unfavorable environmental conditions [53-56]. Pathogenicity of the aforementioned diseases peaks with elevated air temperature and atmospheric humidity [54, 57-59]. Therefore, stressful environmental conditions (e.g., elevated air temperature, drought, etc.) may falsely appear to cause *P. annua* senescence when heightened pathogenetic activity is the actual cause of mortality.

Although the nature of *P. annua* transience in some situations remains uncertain, review of literature indicates the species may be incorrectly botanically characterized [60]. If any environmental factors are causing plant death rather than programmed senescence after inflorescence production, classification as an annual species does not follow the accepted definition of such a life cycle [3]. Given the world-wide presence of the species and difficulties related to management [2, 61], efforts to better understand *P. annua* life cycle are warranted. Therefore, the objective of this study was to determine environmental factors lethal to *P. annua.*

Materials and methods

Life cycle observation

An observational field study was initiated on 11 May 2020 at three locations in Knoxville, TN. One hundred *P. annua* plants were monitored throughout the summer and fall until 27 Oct. 2020. Monitoring occurred in ten unique micro-environments across the three study locations (Table 1). Experimental areas within micro-environments measured 1 x 2 m. Ten *P. annua* plants were identified based on their similar size and growth stage and monitored within each area. Metal rings (7.6 cm in diameter and 2.8 cm in length) were installed around each plant to allow observation of the same *P. annua* plants throughout the study. Herbicides were not applied to the experimental areas during the observational period.

Table 1. Site characteristics for micro-environments monitored from 11 May to 27 October of 2020 to assess *Poa annua* survival.

Number†	GPS coordinates	Height of cut (cm)	Soil series	Soil texture	Soil pH	Soil organic matter (%)
1	36.091N, -83.845W	1.3	Urban land-Udorthents complex	Loam (42% sand, 46% silt, 12% clay)	5.5	5.6
2	36.091N, -83.845W	1.3	Urban land-Udorthents complex	Sand (90% sand, 4% silt, 6% clay)	6.1	2.3
3	36.091N, -83.845W	1.3	Urban land-Udorthents complex	Sandy loam (76% sand, 16% silt, 8% clay)	5.9	3.5
4	36.091N, -83.845W	1.3	Urban land-Udorthents complex	Loam (40% sand, 46% silt, 14% clay)	5.4	7.9
5	36.091N, -83.845W	1.3	Urban land-Udorthents complex	Silt loam (32% sand, 58% silt, 10% clay)	5.8	7.3
6	36.091N, -83.845W	1.3	Urban land-Udorthents complex	Sand (92% sand, 6% silt, 2% clay)	6.1	2.9
7	35.911N, -83.955W	1.6	Waynesboro loam	Silt loam (18% sand, 60% silt, 22% clay)	6.4	6.4
8	35.980N, -84.324W	1.4	Collegedale silt loam	Loamy sand (82% sand, 14% silt, 4% clay)	6.8	5.1
9	35.980N, -84.324W	6.4	Collegedale silt loam	Silt loam (40% sand, 50% silt, 10% clay)	7.2	4.2
10	35.980N, -84.324W	1.4	Collegedale silt loam	Loamy sand (84% sand, 14% silt, 2% clay)	6.6	3.4

† Micro-environments are assigned a number for clarification of results.

At study initiation and every three weeks thereafter, photographs were taken of each *P. annua* plant. Plant mortality was also visually recorded on each rating date and scored as a lack of green tissue aboveground within each metal ring. A multifactor F-score analysis of climatic data related to death events was conducted using Python (Version 3.9.9, https://www.python.org/) programming language. Libraries used in analyses included ‘pandas’ (v. 1.3.4; https://pandas.pydata.org/) for data handling, ‘NumPy’ (v. 1.22.0; https://numpy.org/) for numerical transformations and math, ‘scikit learn’ (v. 1.0.1; https://scikit-learn.org/stable/) for modeling and related functions, ‘SciPy’ (v. 1.7.1; https://scipy.org/) for code modeling and curve fitting, ‘matplotlib’ (v. 3.5.0; https://matplotlib.org/) and seaborn (v. 0.11.2; https://seaborn.pydata.org/) for visualization, and ‘xgboost’ (v. 1.5.1; https://xgboost.readthedocs.io/en/stable/) for broad modeling to determine feature importance. All statistical analyses are available in a Google collaborative environment at: https://colab.research.google.com/drive/16eIDgyFMX08K2szhc_LdXmbYavf982jY?usp=sharing.

Soil temperature evaluation

Ecotypes from micro-environments one and two (Table 1) in the aforementioned observational study were selected on the basis of observed length of life for inclusion in a repeated glasshouse experiment. The experiment was conducted in Knoxville, TN (35.946590, -83.939360) during early spring 2021. Mature *P. annua* plants were harvested from the field on 24 Feb. 2021. Harvest location one was comprised of plants that senesced during 2020, whereas harvest location two contained plants that survived the entire duration of our observational

experiment in 2020. Plants were harvested within a 7.5 m^2 area as undisturbed soil cores (10.2 cm diameter) containing aboveground biomass and an intact root system. Plants of similar size, tiller number, and growth stage (e.g., no inflorescence) were selected for collection. Extracted soil cores were cut to a uniform depth of 7.6 cm, such that no roots were visibly protruding.

Experimental setup and design

Plants were acclimated in a glasshouse for seven days before being placed in water baths to impose variable soil temperatures, similar to the methods used in other experiments investigating soil temperature effects on grass mortality [62-63]. Water baths were constructed by mounting polyvinyl chloride (PVC) pipes (10.2 cm in length and diameter) in plastic containers (51 x 39 x 15 cm) (Fig 1). Pipes were affixed to the container using waterproof caulk atop drainage holes. Three *P. annua* plants from each micro-environment were installed in pipes by sliding the core into the pipe without disturbance. Pipes were randomized within each water bath. Filter paper was placed in the bottom of each tube to prevent loss of soil while allowing for water movement.

Fig 1. *Poa annua* plants harvested on 24 February 2021 in Knoxville, TN placed in polyvinyl chloride pipes (10.2 cm diameter and length) and mounted in plastic tubs. Water added outside of pipes was heated to 21.1, 26.7, 32.2, or 37.8°C to elevate soil temperatures.

Glasshouse conditions averaged 50% relative humidity and 22.8°C air temperature. A 16/8 hour day/night artificial photoperiod was imposed via metal halide lights (1000 W; P.L Light Products, Beamsville, ON, Canada) with night occurring from 2200 to 0600 hours. At the beginning of the acclimation period, plants were supplied with complete fertilizer (20N-20P_2O_5-20K_2O; Southern Ag Triple Twenty with minors; Hendersonville, NC) to deliver 49 kg N ha^{-1}

and immediately irrigated (5 mm) via an overhead sprinkler system. Five mm of irrigation was applied daily between 0700 and 0900 hours. Plants were clipped to a height of 2.5 cm by hand using scissors on days three and seven of acclimation.

After acclimation, plants were subjected to one of four soil temperatures for four weeks. Water baths were arranged in a split-plot design with three replications. The whole plot factor was soil temperature whereas the split-plot factor was *P. annua* ecotype (n = 72 plants; 2 ecotypes x 3 plant replications x 4 soil temperatures x 3 water bath replications). Two experimental runs were conducted simultaneously in separate glasshouse bays.

Aquarium heaters with digital thermometers (500 W; Hygger, Renton, WA) were used to manipulate water temperature in each bath to augment soil temperature. Each water bath was equipped with a 4 W circulation pump and a secondary liquid thermometer to monitor water temperatures. For the duration of this experiment, water bath temperatures were 21.1, 26.7, 32.2, or 37.8°C. Temperatures in this range were chosen because previous studies assessing soil temperature effects on C_3 grasses (such as *P. annua*) consider 20°C and 35°C to be optimal and supraoptimal soil temperatures, respectively, for root growth [62-63].

For water baths set to 26.7, 32.2, or 37.8°C, water was added outside of PVC pipes containing *P. annua* plants to a depth of 7.6 cm. Water baths set to these temperatures were placed on a heating mat (iPower, Irwindale, CA) to prevent heat loss and ensure intended temperatures were achieved. The 21.1°C temperature was achieved using the same equipment devoid of water. Heat from water in baths was transferred to soil cores containing *P. annua* until soil temperature was in equilibrium with water temperature.

Soil temperatures were monitored in soil cores within one temperature replication of each water bath using external soil sensors (#3667; Spectrum Technologies Inc., Aurora, IL)

programmed to record soil temperature every 15 min and transmitted to a micro station (WatchDog 1000 Series; Spectrum Technologies Inc., Aurora, IL). Micro stations also recorded air temperature and humidity every 15 minutes. Secondary digital soil thermometers (#6300; Spectrum Technologies Inc., Aurora, IL) were monitored daily in one replication of each water bath as well. In experimental run one, mean air temperature was 22.6°C and mean relative humidity was 49.4%. In experimental run two, mean air temperature was 22.5°C and mean relative humidity was 46.0%.

Soil temperature treatments were imposed for four weeks. During the treatment period, plants were clipped daily to a height of 2.5 cm by hand using scissors and water baths were refilled to the appropriate water level as needed. Plants were maintained at a constant gravimetric moisture content via addition of supplemental water (15 to 23 mL) twice per week. After four weeks of exposure to soil temperature treatments, plants were subjected to ambient soil temperature for two weeks to assess the potential for regrowth. During this time, plants were not clipped, and supplemental irrigation (5 mm) was applied daily via an overhead misting system.

Data collection and analysis

Plants were visually evaluated daily for mortality, rated as all aboveground tissue necrotic, with death confirmed via assessments of photochemical efficiency using a handheld fluorometer (Fv/Fm meter; Opti-Sciences, Inc., Hudson, NH) to measure Fv/Fm ratio. Two readings were taken from each plant once per week after randomly placing five- to- ten attached leaves into dark adaption chambers for 20 minutes. Plants were considered dead when the Fv/Fm

ratio was zero. For most plant species, Fv/Fm ratios in the range of 0.79 to 0.84 are considered optimum, whereas lower values indicate plant stress [64-65].

Data were subjected to analysis of variance using the SAS (University Edition, SAS, Cary, NC) mixed procedure. Experimental run, soil temperature, and ecotype interactions were tested. Analysis of variance revealed experimental run and ecotype were not significant factors for photochemical efficiency data. Figures were generated in Prism (Prism 9 for Mac, Graph-Pad software, La Jolla, CA). Data are openly available at https://mesurpubshare.blob.core.windows.net/public/PoaLifeCycle_PLOS_Data.xlsx.

Soil water evaluation

Mature *P. annua* plants that survived the entire experiment in 2020 were harvested from micro-environment two (Table 1) on 21 April 2021 for inclusion in a glasshouse experiment exploring effects of soil moisture on *P. annua* mortality at different soil temperatures. Harvest methods were identical to those previously described. All harvested plants were of similar size and had inflorescences present at the time of collection. The collection area was treated with cyazofamid (Segway; PBI Gordon Corporation, Shawnee, KS) at 1.1 kg ha^{-1} 24 hours prior to harvest to prevent confounding effects of soil pathogens. Fungicide was immediately irrigated into the soil after application.

Experimental setup and design

Soil cores containing *P. annua* were tightly wrapped with polyethylene plastic (0.15 mm thick) to prevent water loss via evaporation. *Poa annua* foliage was carefully moved to protrude

out of a 2.5 cm incision at the top of each core (Fig 2). Four drainage holes were created in the bottom of soil cores. Plastic wrapped soil cores were installed in PVC pipes (10.2 cm in length and diameter). Plants then underwent 7 d of acclimation in a glasshouse under conditions described previously for photoperiod, nutrient application, irrigation, and clipping. Ambient conditions during acclimation averaged 64.9% relative humidity and 22.2°C air temperature in experimental run one and 53.9% relative humidity and 22.1°C air temperature in experimental run two.

Fig 2. *Poa annua* plants harvested in soil cores (10.2 cm diameter) wrapped in plastic (0.15 mm) and installed in polyvinyl chloride pipes (10.2 cm diameter and length).

Soil cores were saturated to the point of water freely draining through drainage holes at the termination of the acclimation period. Water was allowed to drain overnight, resulting in soil cores at pot capacity at the onset of treatment; an approach used by other researchers exploring effects of soil water stress on plants [66]. Thirty-six *P. annua* plants in each experimental run were subjected to a 2 x 2 factorial arrangement of soil temperature and irrigation regime for six weeks. Two experimental runs were conducted concurrently in separate glasshouse bays. During treatment, plants received no supplemental nutrients and were clipped daily to a 2.5 cm height using scissors.

Soil temperature was either ambient or elevated via a heating mat (iPower, Irwindale, CA). Irrigation treatments included well-watered (n = 12) or full drought (n = 24; Fig 3). Irrigation needs were determined by calculating daily plant transpiration [66]. Individual PVC pipes were weighed at 1200 to 1300 hours daily. Daily transpiration was calculated as the difference in weight of each pot on successive days. The well-watered plants were irrigated by hand daily to maintain a weight of no less than 50 g of their initial weight. A syringe was

inserted through the incision at the top of the core to facilitate controlled watering. Plants subjected to full drought conditions received no supplemental irrigation throughout the entirety of the study.

Fig 3. *Poa annua* plants harvested on 21 April 2021 in Knoxville, TN and wrapped in 0.15 mm polyethylene plastic to prevent soil water evaporation. Plants were subjected to either ambient or elevated soil temperature via a heating mat. Within each soil temperature treatment, water was either completely withheld or added to meet transpirational loss.

Air temperature, relative humidity, and soil temperature were monitored as previously described. In experimental run one, soil temperature averaged 26.8°C in cores subjected to ambient conditions and 34.6°C in cores subjected to elevated soil temperature. Mean air temperature was 26.6°C and mean relative humidity was 59.4%. In experimental run two, mean soil temperatures were 24.7°C and 32.0°C for cores subjected to ambient and elevated soil temperature, respectively. Mean air temperature was 25.7°C and mean relative humidity was 56.1.%.

Data collection and analysis

Data collection and analyses were similar to those described in the soil temperature experiment. Plants were evaluated daily for mortality on a binary scale where 0 = alive (green tissue observed) and 1 = dead (all tissue necrotic). Mortality was confirmed via photochemical efficiency readings. *Poa annua* photochemical efficiency was evaluated three times per week by measuring chlorophyll fluorescence using a handheld fluorometer (Fv/Fm meter; Opti-Sciences, Inc., Hudson, NH). Two readings were taken from each plant after randomly placing five- to- ten attached leaves into dark adaption chambers for 20 minutes. Volumetric water content (VWC) of soil was recorded at approximately a 5.0 cm depth on the day of mortality, or for those that

survived, at the conclusion of the study by inserting a soil moisture sensor (ML3 ThetaProbe; Delta -T Devices, Cambridge, UK) into the center of each core and recording a single reading.

Visually rated mortality data were subjected to life cycle analysis in Prism (Prism 9 for Mac, Graph-Pad software, La Jolla, CA) using the Kaplan-Meier estimate of survival probabilities log rank test with $P < 0.0001$ determining differences in mortality. Data are openly available at https://mesurpubshare.blob.core.windows.net/public/PoaLifeCycle_PLOS_Data.xlsx.

Disease susceptibility evaluation

A field experiment was conducted at the East Tennessee AgResearch and Education Center (Knoxville, TN) from 27 May to 28 October 2021 to assess the impacts of fungicide application on *P. annua* mortality. The experimental site was a mixed stand of *P. annua* and zoysiagrass (*Zoysia matrella* Merr. cv. Trinity) atop a Sequatchie silt loam soil with a pH of 6.2 and 2.9% organic matter. The area was mown twice per week at a 1.3 cm height of cut and watered four times per week via automatic irrigation. Slow-release fertilizer (Gal-Xe$^{ONE®}$; Simplot, Boise, ID) was applied to the entire area on 22 April 2021 to deliver 195 kg N ha^{-1} yr^{-1}. On 25 May 2021, the experimental area was treated with quinclorac (Drive XLR8; BASF Corporation, Research Triangle Park, NC) at 0.84 kg ha^{-1} + methylated seed oil (0.5% v/v) to control summer annual weeds.

The experiment was arranged as a randomized complete block design with 10 replications of two treatments applied to 1 m^2 plots. Treatments included either no fungicide application or fungicide mixtures including active ingredients from Fungicide Resistance Action Committee groups #2, 3, 5, 7, 11, and 21 intended to manage outbreaks of dollar spot,

anthracnose, *Pythium* blight, and brown patch (Table S1). Treatments were applied every 14 days from 26 May to 13 October 2021. Every two weeks, experimental units were visually assessed for *P. annua* mortality. An F-score analysis of climatic data associated with mortality was conducted using the same methods and packages as those used in the life cycle monitoring experiment.

Results & discussion

Life cycle observation

In four of the 10 micro-environments (#2, #6, #7, and #9) all ten monitored *P. annua* plants survived the summer season, whereas in the other six micro-environments, all ten monitored plants senesced during the observational period. The ability of *P. annua* to survive year-round in certain environments in this study aligns with reports of perennial ecotypes [6-30].

Surviving plants exhibited a range of morphological characteristics. Plants from locations two and six were fine-textured, displayed lateral growth, and produced inflorescences periodically throughout summer (Fig S1). Plants in locations seven and nine were coarse-textured with an upright growth habit and lacked inflorescences. Micro-environments conducive to *P. annua* survival were those where water was either not a limiting factor or where fungicides were applied. Analysis of environmental factors at the time of observed plant mortality corroborated these observations and indicated that sum precipitation, maximum soil temperature, and likelihood of pathogenic infection were significant factors related to plant death (Fig 4).

Fig 4. F-score analysis conducted in Python of climatic data related to death events of *Poa annua* monitored for length of life from May to October 2020 in ten micro-environments in Knoxville, TN.

Soil temperature evaluation

Analysis of variance revealed only a significant soil temperature treatment effect in photochemical efficiency data. Therefore, data were pooled across experimental runs and ecotypes. Considering that ecotypes in this experiment were harvested from distinct microenvironments where we observed mortality or continued survival in our observational study, the lack of an ecotype effect in photochemical efficiency provides evidence that *P. annua* mortality is dictated by environmental conditions rather than programmed senescence common of annual plants.

Poa annua plants survived 28 days of exposure to soil temperatures of 21.1, 26.7, or 32.2°C and senesced when subjected to 37.8°C. Plants subjected to the 21.1°C soil temperature remained healthy for the duration of the experiment as photochemical efficiency was near optimum (Fv/Fm of 0.79), ranging from 0.75 to 0.78 (Fig 5). Conversely, plants subjected to the 37.8°C soil temperature exhibited drastically reduced photochemical efficiency from 7 DAT until the end of the study. *Poa annua* plants exposed to the 26.7 or 32.2°C soil temperature treatments did not senesce but were negatively affected by prolonged exposure to elevated soil temperatures; both treatments numerically reduced photochemical efficiency from 21 to 42 days after treatment (DAT). For plants subjected to the 37.8°C soil temperature, photochemical efficiency was drastically reduced from 0.71 at 7 DAT to 0.33 by 14 DAT, remaining ≤ 0.02 thereafter, indicating death.

Fig 5. Photochemical efficiency was evaluated by measuring chlorophyll fluorescence (Fv/Fm) twice per *Poa annua* plant using a fluorometer. Measurements were taken once

per week from plants subjected to soil temperatures of 21.1, 26.7, 32.2, or 37.8°C imposed via water baths. The dotted line indicates optimal chlorophyll fluorescence of 0.79.

Only the 37.8°C soil temperature treatment was lethal to *P. annua* plants with mortality first noted 12 DAT. By 17 DAT, 35 of 36 *P. annua* plants exposed to the 37.8°C soil temperature had died. Reductions in *P. annua* plant health without death, except in extreme cases, when soil temperature is elevated supports other published reports [32 ,51]. For example, exposure of 115 *P. annua* selections to conditions of 47°C at 100% relative humidity in a growth chamber for six hours did not cause mortality, however 77% of plants were negatively affected (< 50% recovery) [32]. Similarly, *P. annua* subjected to day/night air temperatures of 20/15, 30/25, or 40/35°C for eight days in growth chambers resulted in physiological damage only from the supraoptimal temperature of 40/35°C, although no plants senesced [51].

Survival of *P. annua* in these glasshouse experiments agreed with observations made in our life cycle monitoring experiment in the field. For example, peak soil temperature across all ten micro-environments was 30.8°C (The Earthstream Platform; mesur.io, Yanceyville, NC) in 2020 and average daily air temperature did not exceed 28.7°C. Given that some *P. annua* plants in the observational experiment senesced without soil temperatures exceeding 32.2°C, we concluded that elevated soil temperature alone does not result in *P. annua* death.

Soil water evaluation

A log-rank test detected significant (P <0.0001) differences in survival probability among treatments. Survival probability for plants subjected to the well-watered + ambient soil temperature treatment was 100% throughout the experiment (Fig 6). No treatment resulted in a survival probability of 0% during the tested time frame. However, across all treatments, 27 of 72

tested *P. annua* plants did senesce. The lowest *P. annua* survival probability was observed for the full drought + elevated soil temperature treatment; Probability of survival was not reduced from 100% until 19 DAT and remained above 50% through 40 DAT. Comparatively, survival probability for plants exposed to the well-watered + elevated soil temperature and full drought + ambient temperature treatments was 100% through 34 DAT and remained above 70% through 42 DAT.

Fig 6. Plot of survival probabilities for *Poa annua* plants maintained at ambient or elevated soil temperatures and subjected to either well-watered or full drought conditions for 42 days. Probabilities were generated using the Kaplan-Meier estimate of survival function in Prism.

The ability of *P. annua* to survive an extended period with low water availability is documented [50]. These researchers did not observe *P. annua* senescence when plants were maintained in a sand-based medium with VWC from 4 to 12% for 95 days. While VWC treatments tested are similar to those achieved via drought stress treatment (6.6 to 8.4%) in this study, air and soil temperature were not published in their report [50]. Although a combination of full drought and elevated soil temperature was lethal to some *P. annua* plants in our experiment, it is telling that the probability of *P. annua* survival was still 25% after 42 days of continuous exposure to conditions that rarely occur in the field. Moreover, no other treatment combination in this experiment reduced the probability of *P. annua* survival to < 70% during the study. These responses indicate that interactions of soil moisture and temperature are not lethal to *P. annua* except in extreme situations atypical of managed urban grasslands.

Disease susceptibility evaluation

Poa annua plants in plots not treated with fungicide did not survive the summer period. High individual plant mortality was observed in non-treated areas on 24 June 2021 with some plots completely devoid of *P. annua* beginning on 8 July 2021 (Fig 7). By 4 August 2021, no *P. annua* plants were alive in any non-treated plots. Conversely, in plots treated with fungicide, some *P. annua* plants survived to the termination of the experiment.

Fig 7. *Poa annua* mortality following either no fungicide treatment or fungicide treatment every 14 days from 27 May to 28 October 2021. The y-axis represents anthracnose (*Colletotrichum cereale*) severity index calculated using the Danneberger model [53], where an anthracnose severity index of 2 is the minimum conditions for infection. The x-axis represents time, indicated by month of year. Within each month, *Poa annua* mortality is split by fungicide treatment with vertical space equating to the range of anthracnose severity across the month and horizontal space equating to the smoothed number of senescence observations corresponding to each treatment. The figure was created in Python using 'matplotlib' (v. 3.5.0; https://matplotlib.org/) and seaborn (v. 0.11.2)

The range of the anthracnose caused by *C. cereale* severity (Fig 7) in this experiment suggests that increases in zoysiagrass cover throughout the monitoring period (and a concomitant decline in *P. annua* plant size) may have affected our ability to document *P. annua* during summer. For example, zoysiagrass cover across the experimental area at study initiation was only 63%, whereas by 18 August 2021, zoysiagrass cover reached 88%. Zoysiagrass is a C_4 species adapted to the summer season in Knoxville, TN and *P. annua* is a C_3 species that is at a disadvantage during summer. Therefore, we surmised that zoysiagrass outcompeted *P. annua* for resources and caused a natural population dynamic shift. Inter-species competition with common bermudagrass (*Cynodon dactylon* Pers.) has limited *P. annua* persistence in summer months

previously [8]. As zoysiagrass grew denser, *P. annua* became more difficult to assess via visual ratings. Without the presence of an inflorescence to clearly identify *P. annua* plants, it is possible plants were not identified, but persisted throughout the season (Fig 8). For example, fungicide treated plots scored as not containing *P. annua* on 4 August contained multi-tillered plants in autumn when temperatures cooled and suggest that the roots and underground shoots of the plants had survived the summer and grew rapidly when favorable environmental conditions for growth were restored.

Fig 8. *Poa annua* plants with inflorescences on 4 August 2021 following biweekly fungicide applications in a zoysiagrass (*Zoysia matrella* Merr. cv. 'Trinity') urban grassland located in Knoxville, TN. Without the presence of inflorescences, plants would likely be difficult to identify.

Interestingly, several *P. annua* plants maintained vigorous growth throughout the experiment, producing inflorescences as early as 4 August 2021 (Fig 8). *Poa annua* emergence models developed in Knoxville, TN indicate peak emergence typically occurs between the 40th and 43rd week of the calendar year when mean seven-day soil temperature lowers to ≤ 18.9ºC and seven-day rainfall accumulation is ≥ 12.7 mm [67]. Observation of *P. annua* inflorescence production in only the 32nd week of the year when soil temperatures were > 24 ºC and average weekly rainfall was < 4.75 mm (The Earthstream Platform; mesur.io, Yanceyville, NC) supports our assertion that observed plants were not newly germinated but had survived the summer season.

Results of this experiment indicate that fungal pathogens are the primary cause of *P. annua* mortality in maintained urban grasslands. Although not all plants in fungicide treated plots survived or were observable, *P. annua* plants treated with fungicide lived markedly longer than

those not treated with fungicide (Fig 7). F-score analysis of climatic data at the time of plant mortality revealed risk of anthracnose and brown patch infection as significant factors associated with mortality (Fig 9). On the first date mortality was documented in non-treated plots (24 June 2020), anthracnose acervuli were present on necrotic foliage.

Fig 9. F-score analysis conducted in Python of climatic data related to death events of *Poa annua* plants monitored for length of life from May to October 2021 in a zoysiagrass (*Zoysia matrella* Merr., cv. Trinity) fairway. Plants were either left without treatment or treated with fungicide mixtures intended to control dollar spot (*Clarireedia* spp.), anthracnose (*Colletotrichum cereale*), Pythium blight (*Pythium* spp.), and brown patch (*Rhizoctonia solani*) every 14 days.

Summer decline of *P. annua* via fungal infection is thoroughly discussed in pathology literature [52, 58, 68]. Fungicide use on *P. annua* managed as a desirable species is a readily accepted management practice for summer survival [69-70]. Some reports indicate *P. annua* cannot be commercially released until disease resistance is incorporated into the species because plants are so highly susceptible to pathogen attack [8]. Therefore, we recommend that susceptibility to fungal pathogens be considered when assessing the life cycle of *P. annua*.

Conclusions

Results of these experiments suggest *P. annua* perishes from fungal infection, which may be exacerbated by environmental and anthropogenic stresses during summer months [56, 57, 59]. *Poa annua* seems to persist unless environmental conditions are unfavorable, often presenting as a polycarpic plant rather than succumbing to programmed senescence. This environmentally driven response is similar to that of other perennial C_3 grass species such as Kentucky bluegrass

(*Poa pratensis* L.) or tall fescue (*Festuca arundinace*[illegible]chreb.). On the contrary, annual species are monocarpic plants senescing after a single reproductive cycle regardless of environment [3]. Our results show that *P. annua* does not meet the definition of an annual species. Observations made in this study and via an exhaustive review of peer-reviewed literature [60] present little evidence supporting an inherently annual life cycle in *P. annua*. We contend the epithet "annua" is a misnomer according to its current interpretation. Although "annual" can be associated with life cycle, "occurring once every year" is also a definition of the word [71]. Thus, the species epithet "annua" may have been awarded to *P. annua* to mark a yearly observation such as growth at the same location or yearly inflorescence production rather than of an annual life cycle.

Early taxonomic descriptions of *P. annua* provide no evidence of life cycle study at the time of naming, indicating the species name has been misconstrued in modern times [60]. The shift in present understanding of *P. annua* as a perennial rather than the long purported annual species has major implications for plant management. Therefore, a more appropriate and descriptive name may be *P. typica* or *P. vulgari*, meaning 'typical' and 'common' in Latin, respectively. These epithets preclude misinformation surrounding life cycle and indicate the species prevalence given that *P. annua* is found on all seven continents in a myriad of climates.

Acknowledgements

Authors thank Drs. Kellie Walters and John Zobel for their assistance in developing methods. Authors would also like to recognize the contributions made by Javier Vargas, Gregory Breeden, and Benjamin Pritchard in experiment set up and management.

Competing Interests

The authors have declared that no competing interests exist.

Financial Disclosure

The authors received no specific funding for this work.

Ethics Statement

Field research and sample harvesting was conducted on university property, Three Ridges Golf Course, or Oak Ridge Country Club. No permits were required for testing or plant collection. Permission for use was granted by site managers.

References

1. Pate JS, Hopper SD. Rare and common plants in ecosystems, with special reference to the south-west Australian flora. In Schulze, E-D. & Mooney, H.A. (Eds.), Biodiversity and Ecosystem Function. Springer-Verlag Berlin Heidelberg.1994.
2. Van Wychen L. Survey of the Most Common and Troublesome Weeds in Grass Crops, Pasture and Turf in the United States and Canada. Weed Science Society of America National Weed Survey Dataset 2020. [cited 23 July 2021]. Available from: https://wssa.net/wssa/weed/surveys/.
3. Taiz L, Zeiger E. Plant Physiology, 3rd Edition. Sinauer Associates. 2002.
4. United States Department of Agriculture. *Zea Mays* L. [cited 27 July 202]. Available from: https://plants.usda.gov/home/plantProfile?symbol=ZEMA
5. United States Department of Agriculture. *Triticum aestivum* L. [cited 27 July 2021]. Available from: https://plants.usda.gov/home/plantProfile?symbol=TRAE
6. Piper CV, Oakley RA. Annual Bluegrass (*Poa annua*). United States Golf Association Green Section Bulletin. 1927;7(7): 128-129.
7. Tutin TG. A contribution to the experimental taxonomy of *Poa annua* L. Watsonia. 1957;4(1).
8. Younger VB. Ecological studies on *Poa annua* in turfgrass. Grass and Forage Sci. 1959;14: 233-237.
9. Timm G. Biology and systematics of *Poa annua*. Feitschrift fr Acker-und Pflanzenbau. 1965;22(3): 267-294.
10. Ellis WM, Lee BTO, Calder, DM. A biometric analysis of populations of *Poa annua* L. Society for the Study of Evolution. 1971;25(1): 29-37. doi:10.2307/2406497

11. Gibeault VA. Perenniality in *Poa annua* L. Doctoral Thesis, Oregon State University. 1971.
12. Bogart JE. Factors Influencing Competition of Annual Bluegrass (*Poa annua* L.) Within Established Turfgrass Communities and Seedling Stands. M.Sc.Thesis, Michigan State University. 1972.
13. Cordukes WE. Growth habit and heat tolerance of a collection of *Poa annua* Plants in Canada. Canadian Journal of Plant Science. 1977;57: 1201-1203. doi:10.4141/cjps77-177
14. Law R, Bradshaw AD, Putwain PD. Life-history variation in *Poa annua*. Evolution. 1977;31(2): 233-246.
15. Warwick SI. The biology of Canadian weeds. 37. *Poa annua* L. Canadian Journal of Plant Sci. 1979;59: 1053-1066. doi:10.4141/cjps79-165
16. Danneberger TK, Vargas JM. Annual bluegrass seedhead emergence as predicted by degree-day accumulation. Agronomy Journal. 1984;76(5): 756-758. doi:10.2134/agronj1984.00021962007600050021x
17. Lush ML. Biology of Poa annua in a temperate zone golf putting green (*Agrostis stolonifera/Poa annua*). I. The above-ground population. Journal of Applied Ecology. 1988;25(3): 977-988.
18. Lush ML. Adaptation and differentiation of golf course populations of annual bluegrass (*Poa annua*). Weed Sci. 1989;37(1): 54-59.
19. Till-Bottraud I, Wu L, Harding J. Rapid evolution of life history traits in populations of *Poa annua* L. Journal of Evolutionary Biology. 1990;3: 205-224. doi:10.1046/j.1420-9101.1990.3030205.x

20. Darmency H, Berti A, Gasquez J, Matjicek A. Association of esterase isozymes with morphology in F_2 progenies of two growth variants in *Poa annua* L. The New Phytologist. 1992;121(4): 657-661. doi:10.1111/j.1469-8137.1992.tb01137.x
21. Johnson PG, White DB. Vernalization requirements among selected genotypes of annual bluegrass (*Poa annua* L.). *Crop Sci.* 1997;37: 1538-1542. doi:10.2135/cropsci1997.0011183X003700050021x
22. Johnson PG, White DB. Flowering responses of selected annual bluegrass genotypes under different photoperiod and cold treatments. Crop Sci. 1997;37: 1543-1547. doi:10.2135/cropsci1997.0011183X003700050022x
23. Mitich LW. Annual Bluegrass (*Poa annua* L.) Weed Technology. 1998;12(2): 414-416. doi:10.1017/S0890037X00044031
24. Cline VW. Population Dynamics of *Poa annua* L. on a Northern Golf Course. Doctoral Thesis, The University of Minnesota. 2001.
25. McElroy JS, Walker RH, van Santen E. Patterns of variation in *Poa annua* populations as revealed by canonical discriminant analysis of life history traits. *Crop Sci.* 2002;42: 513-517. doi:10.2135/cropsci2002.5130
26. McElroy JS, Walker RH, Wehtje GR, van Santen E. Annual bluegrass (*Poa annua*) populations exhibit variation in germination response to temperature, photoperiod, and fenarimol. Weed Sci. 2004;52: 47-52.
27. Stoy AN. Life history traits in *Poa annua* L. populations throughout Utah's diverse environments. M.Sc. Thesis, Utah State University. 2005.
28. Kaminski JE, Dernoeden PH. Seasonal *Poa annua* L. seedling emergence patterns in Maryland. Crop Sci. 2007;47(2): 775-781. doi:10.2135/cropsci2006.03.0191

29. La Mantia JM, Huff DR. Instability of the greens-type phenotype in *Poa annua* L. Crop Sci. 2011;51: 1784-1792. doi:10.2135/cropsci2010.10.0580

30. Mao Q, Huff DR. Characterizing small RNA profiles in allotetraploid *Poa annua* L. and its diploid parents. Crop Sci. 2017;57.S1; S–13–S –25. doi:10.2135/cropsci2016.06.0462

31. Linné C. Species plantarum. 1753; pp 68.

32. Linné C. Herbationes upsalienses. 1754; pp 14.

33. Law R. The dynamics of a colonizing population of *Poa annua*. Ecology. 1981;62(5): 1267-1277.

34. Struik GJ. Growth patterns of some native annual and perennial herbs in Wisconsin. *Ecology*. 1965;46(4): 401-420.

35. Harper JL, Lovell PH, Moore KG. The shapes and sizes of seeds. Annual Review of Ecology and Systematics. 1970;1: 327-356.

36. Medeiros RB, Steiner JJ. White clover seed production: III. Cultivar differences under contrasting management practices. Crop Sci. 2000;40(5): 1317-1324. doi:10.2135/cropsci2000.4051317x

37. Honek A, Martinkova Z, Saska P. Post-dispersal predation of *Taraxacum officinale* (dandelion) seed. Journal of Ecology. 2005;93(2):345-352. doi:10.1111/j.1365-2745.2005.00987.x

38. Campbell BD, Grime JP. An experimental test of plant strategy theory. Ecology. 1992;73(1): 15-29.

39. Williams GC. Pleiotropy, natural selection, and the evolution of senescence. Evolution. 1957;11(4): 398-411.

40. Silvertown JW. Why are biennials sometimes not so few? The American Naturalist. 1983;121(3): 448-453.

41. Iwasa Y, Cohen D. Optimal growth schedule of a perennial plant. The American Naturalist. 1989;133(4): 480-505.

42. Molina-Montenegra MA, Carrasco-Urra F, Acuña-Rodríguez I, Oses R, Torres-Días C, Chwedorzewska KJ. Assessing the importance of human activities for the establishment of the invasive *Poa annua* in Antarctica. Polar Research Journal. 2014;33(1): 21425. doi:10.3402/polar.v33.21425

43. Hobbs WH. The Eurasian continental glacier of the Late Pleistocene. Science. 1946;104(2692): 105-106. doi:10.1126/science.104.2692.105

44. Mao Q, Huff DR. The evolutionary origin of *Poa annua* L. Crop Sci. 2012;52(4): 1910-1922. doi:0.2135/cropsci2012.01.0016

45. Johnson PG. Genetics and physiology of flowering in *Poa annua* L. Doctoral Thesis, University of Minnesota. 1995.

46. Sukumaran NP, Weiser CJ. Freezing injury in potato leaves. Plant Physiology. 1972;50: 564-567.

47. Naddem K, Munawar M, Chishti SAS. Genetic architecture and association of fruit yield and quality traits in tomato (*Solanum lycopersicum* L.). Universal Journal of Agricultural Research. 2013;1(4): 155-159.

48. Yang S, Kaggwa RJ, Andrade-Sanchez P, Zarnstorff M, Wang G. Lint yield compensatory response to main stem node removal in upland cotton (*Gossypium hirsutum*). Journal of Agronomy and Crop Sci. 2015;202(3): 243-253. doi:10.1111/jac.12142

49. Gaussoin RE, Branham BE. Influence of cultural factors on species dominance in a mixed stand of annual bluegrass/creeping bentgrass. Crop Sci. 1989;29: 480-484.
50. Slavens MR, Johnson PG, Bugbee B. Irrigation frequency differentially alters vegetative growth and seed head development of *Poa annua* L. biotypes. Crop Sci. 2011;51: 314-322. doi:10.2135/cropsci2010.01.0006
51. Yang Z, Miao Y, Yu J, Liu J, Huang B. Differential growth and physiological responses to heat stress between two annual and two perennial cool-season turfgrasses. Scientia Horticulturae. 2014;170: 75-81. doi:10.1016/j.scienta.2014.02.005
52. Smiley RW, Dernoeden PH, Clarke BB. Compendium of Turfgrass Diseases Third Edition. The American Phytopathological Society. 2005.
53. Danneberger TK, Vargas JM, Jones AL. A model for weather-based forecasting of anthracnose on annual bluegrass. Phytopathology. 1984;74(4): 448-451.
54. Walsh B, Ikeda SS, Boland GJ Biology and management of dollar spot (*Sclerotinia homeocarpa*); an important disease of turfgrass. *HortSci.* 1999;34(1): 13-21.
55. Inguagiato JC, Murphy JA, Clarke BB. Anthracnose disease and annual bluegrass putting green performance affected by mowing practices and lightweight rolling. Crop Sci. 2009;49(4): 1454-1462. doi:10.2135/cropsci2008.07.0435
56. Roberts JA, Inguagiato JC, Clarke BB, Murphy JA. Irrigation quantity effects on anthracnose disease of annual bluegrass. Crop Sci. 2011;51(3), 1244-1252. doi:10.2135/cropsci2010.07.0444
57. Fidanza MA, Dernoeden PH, Grybauskas AP. Development and field validation of a brown patch warning model for perennial ryegrass turf. Phytopathology. 1996;86(4): 385-340.

58. Inguagiato JC, Martin SB. Disease of Cool- and Warm-Season Putting Greens. United States Golf Association Green Section Record. 2015;53(9).

59. Smith DL, Kerns JP, Walker NR, Payne AF, Horvath B, Inguagiato JC, et al. Development and validation of a weather-based warning system to advise fungicide applications to control dollar spot on turfgrass. PLOS ONE. 2018;13(3): e0194216. doi:10.1371/journal.pone.0194216

60. Carroll DE, Brosnan JT, Trigiano RN, Horvath BJ, Shekoofa A, Mueller TC. Current understanding of the *Poa annua* life cycle. Crop Science. 2021;61(3): 1527-1537. doi:10.1002/csc2.20441

61. Brosnan JT, Elmore MT, Bagavathiannan M. Herbicide-resistant weeds in turfgrass: Current status and emerging threats. Weed Technology. 2020;34: 424-430. doi:10.1017/wet.2020.29

62. Huang B, Xu Q. Root growth and nutrient element status of creeping bentgrass cultivars differing in heat tolerance as influenced by supraoptimal shoot and root temperatures. Journal of Plant Nutrition. 2000;23(7): 979-990. doi:10.1080/01904160009382075

63. Huang B, Liu X, Xu Q. Supraoptimal soil temperatures induced oxidative stress in leaves of creeping bentgrass cultivars differing in heat tolerance. Crop Sci. 2001;41: 430-435.

64. Maxwell K, Johnson GN. Chlorophyll fluorescence – a practical guide. Journal of Experimental Botany. 2000;51(345): 659-668. doi:10.1093/jexbot/51.345.659

65. Wu C, Varanasi V, Perez-Jones A. A nondestructive leaf-desk assay for rapid diagnosis of weed resistance to multiple herbicides. Weed Sci. 2021;69: 274-283. doi:10.1017/wsc.2021.15

66. Sheldon K, Shekoofa A, Walker E, Kelly H. Physiological screening for drought-tolerance traits among hemp (*Cannabis sativa* L.) cultivars in controlled environments and in field. Journal of Crop Improvement. 2021;35(6): 816-831. doi:10.1080/15427528.2021.1883175
67. Taylor DR, Prorock M, Horvath BJ, Brosnan JT. Modeling seasonal emergence of *Poa annua* in urban greenspace. Scientific Reports. 2021;11: 18960. doi:10.1038/s41598-021-9825-4
68. Agnew ML. Building disease control programs on annual bluegrass greens in the mid-Atlantic region. Golf Course Management magazine. 2007;75: 91-97.
69. Gelernter WD, Sotwell LJ, Johnson ME, Brown CD. Documenting trends in pest management practices on US golf courses. Crop, Forage & Turfgrass Management. 2016;2(1): 1-9. doi:10.2134/cftm2016.04.0032
70. Lyman GT, Johnson ME, Stacey GA, Brown CD. Golf course environmental profile measures pesticide use practices and trends. Applied Turfgrass Sci. 2012. doi:10.1094/ATS-2012-1220-01-RS.
71. Merriam-Webster. Annual definition and meaning. n.d. Last accessed 11 May 2022 from https://www.merriam-webster.com/dictionary/annual

Supporting information

S1 Figure. ***Poa annua*** **L. plants of wide morphological variation in texture and growth habit observed to survive from 11 May to 27 October 2020 in Knoxville, TN. Images taken on 4 August 2020 where A = a fine-texture, laterally growing plant with inflorescence in micro-environment two; B = a fine-texture, laterally growing plant in micro-environment six; C = an upright growing, coarse textured plant in micro-environment seven; and D = an upright growing, coarse textured plant in micro-environment nine.**

S1 Table. Fungicide applications made between May and October 2021 to control diseases common to ***Poa annua.***

Application date	Fungicide trade name	Producing company (brand, city, state)	Active ingredient rate (ha^{-1})	FRAC† mode of action group number
27 May	Heritage Action	Syngenta, Research Triangle Park, NC	0.3 kg azoxystrobin	11
	Posterity	Syngenta, Research Triangle Park, NC	0.05 kg pydifluometofen	7
10 June	Maxtima	BASF Corporation, Research Triangle Park, NC	0.76 kg mefentrifluconazole	3
	Chipco Signature	Bayer Environmental Science, Cary, NC	9.8 kg aluminum tris	2
24 June	Daconil Ultrex	Syngenta, Research Triangle Park, NC	9.2 kg clorothalonil	5
	Segway	PBI Gordon Corporation, Shawnee, KS	0.72 kg cyazofamid	21
8 July	Insignia	BASF Corporation, Research Triangle Park, NC	0.71 kg pyraclostrobin	11
	Chipco Signature	Bayer Environmental Science, Cary, NC	9.8 kg aluminum tris	2
22 July	Ascernity	Syngenta, Research Triangle Park, NC	0.07 kg benzovindiflupyr + 0.25 kg difenoconazole	7
	Segway	PBI Gordon Corporation, Shawnee, KS	0.72 kg cyazofamid	21
4 Aug	Chipco Signature	Bayer Environmental Science, Cary, NC	9.8 kg aluminum tris	2
	Rayora	FMC, Philadelphia, PA	0.6 kg flutriafol	3
18 Aug	Heritage Action	Syngenta, Research Triangle, NC	0.3 kg azoxystrobin	11
	Daconil Ultrex	Syngenta, Research Triangle Park, NC	9.2 kg clorothalonil	5
	Segway	PBI Gordon Corporation, Shawnee, KS	0.72 kg cyazofamid	21
2 Sep	Maxtima	BASF Corporation, Research Triangle Park, NC	0.76 kg mefentrifluconazole	3
	Chipco Signature	Bayer Environmental Science, Cary, NC	9.8 kg aluminum tris	2
16 Sep	Insignia	BASF Corporation, Research Triangle Park, NC	0.71 kg pyraclostrobin	11
	Posterity	Syngenta, Research Triangle, NC	0.05 kg pydifluometofen	7
30 Sep	Maxtima	BASF Corporation, Research Triangle Park, NC	0.76 kg mefentrifluconazole	3
	Segway	PBI Gordon Corporation, Shawnee, KS	0.72 kg cyazofamid	21
13 Oct	Daconil Ultrex	Syngenta, Research Triangle Park, NC	9.2 kg clorothalonil	5
	Heritage Action	Syngenta, Research Triangle, NC	0.3 kg azoxystrobin	11

† Fungicide Resistance Action Committee (FRAC)

Supplemental Figure 1

Click here to access/download;Figure;S1_Figure.tiff

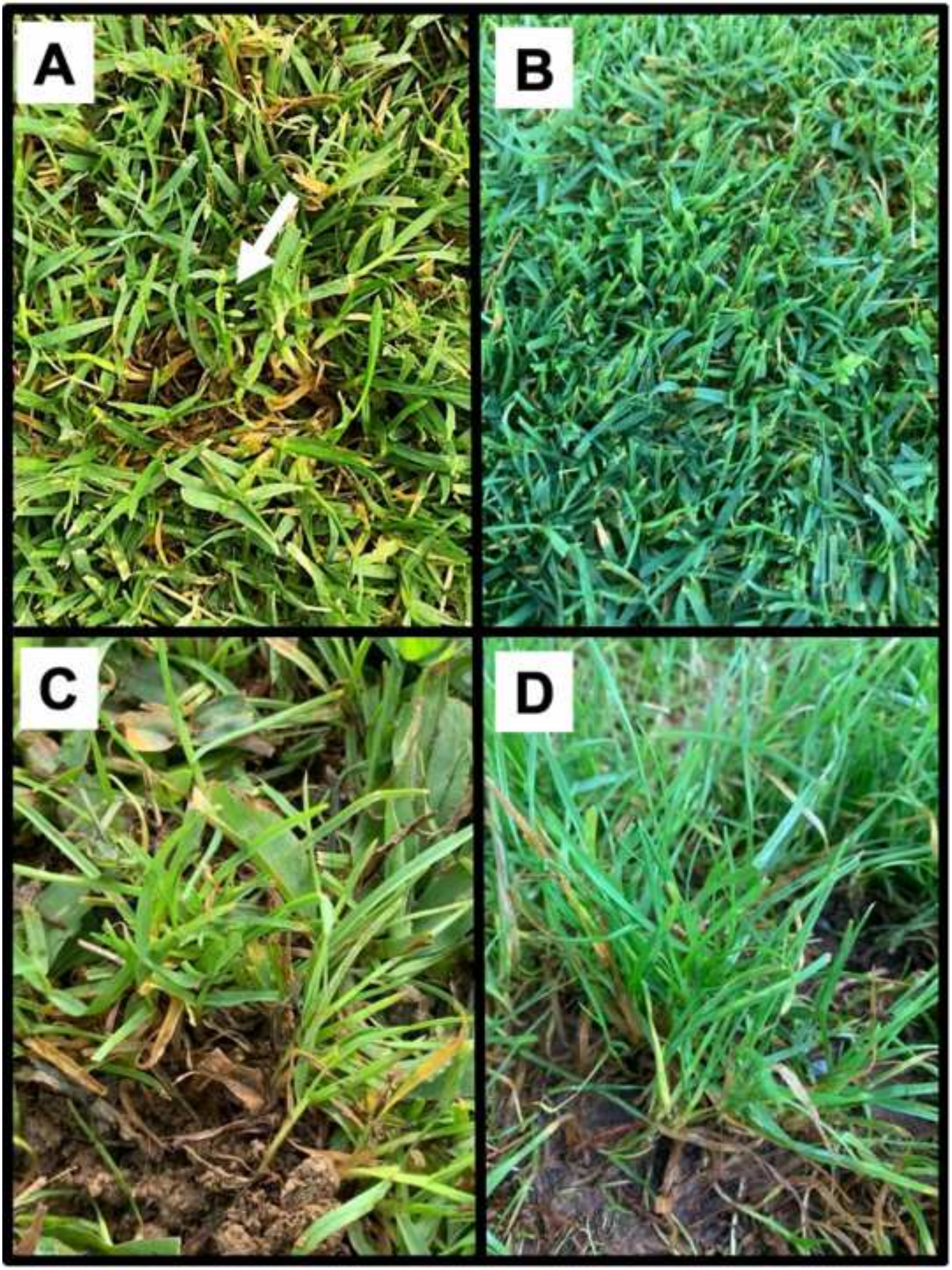

Figure 1

Click here to access/download;Figure;Fig1.tiff

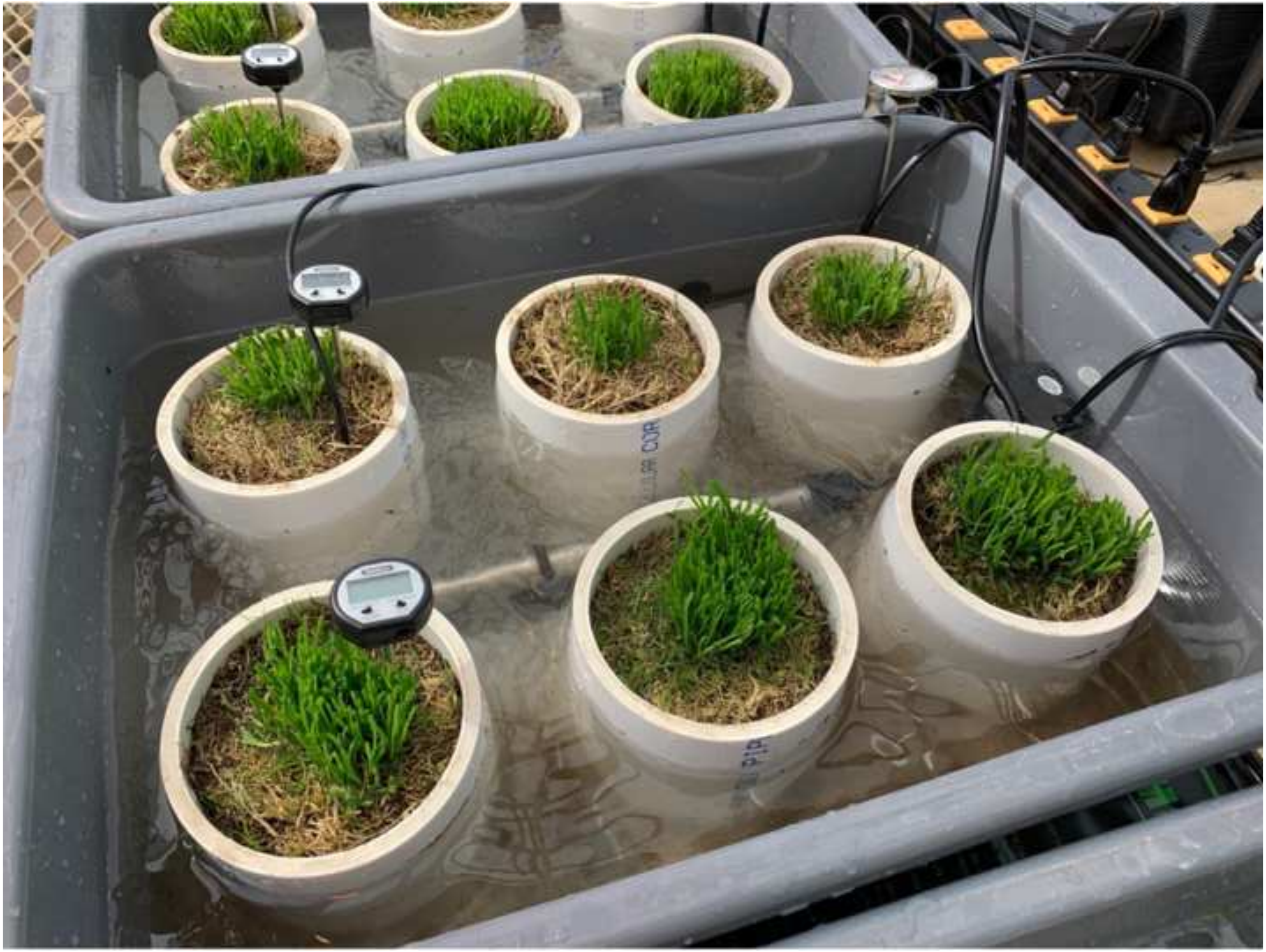

Figure 2

Click here to access/download;Figure;Fig2.tiff

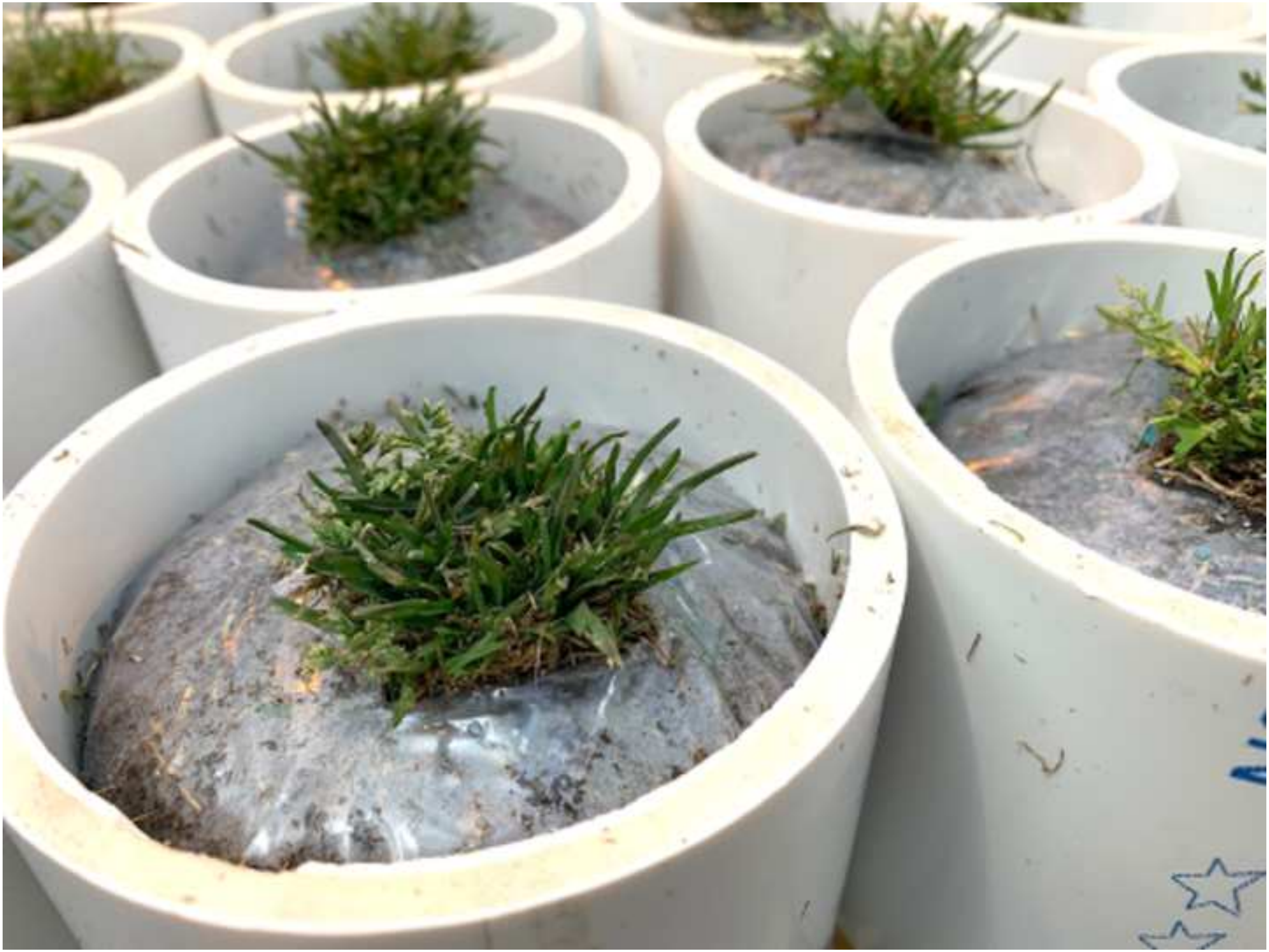

Figure 3

Click here to access/download;Figure;Fig3.tiff

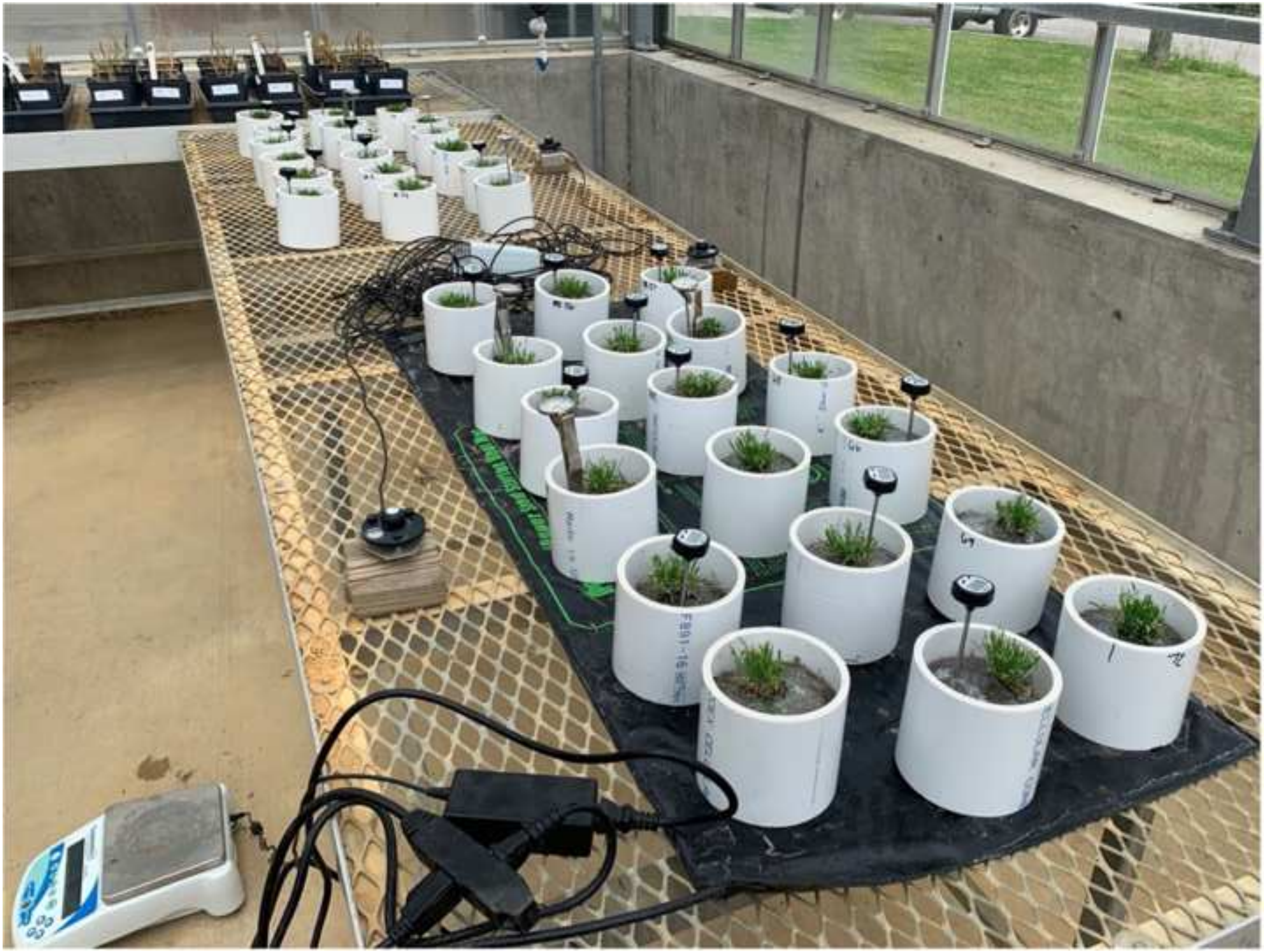

Figure 4

Click here to access/download;Figure;Fig4.tiff

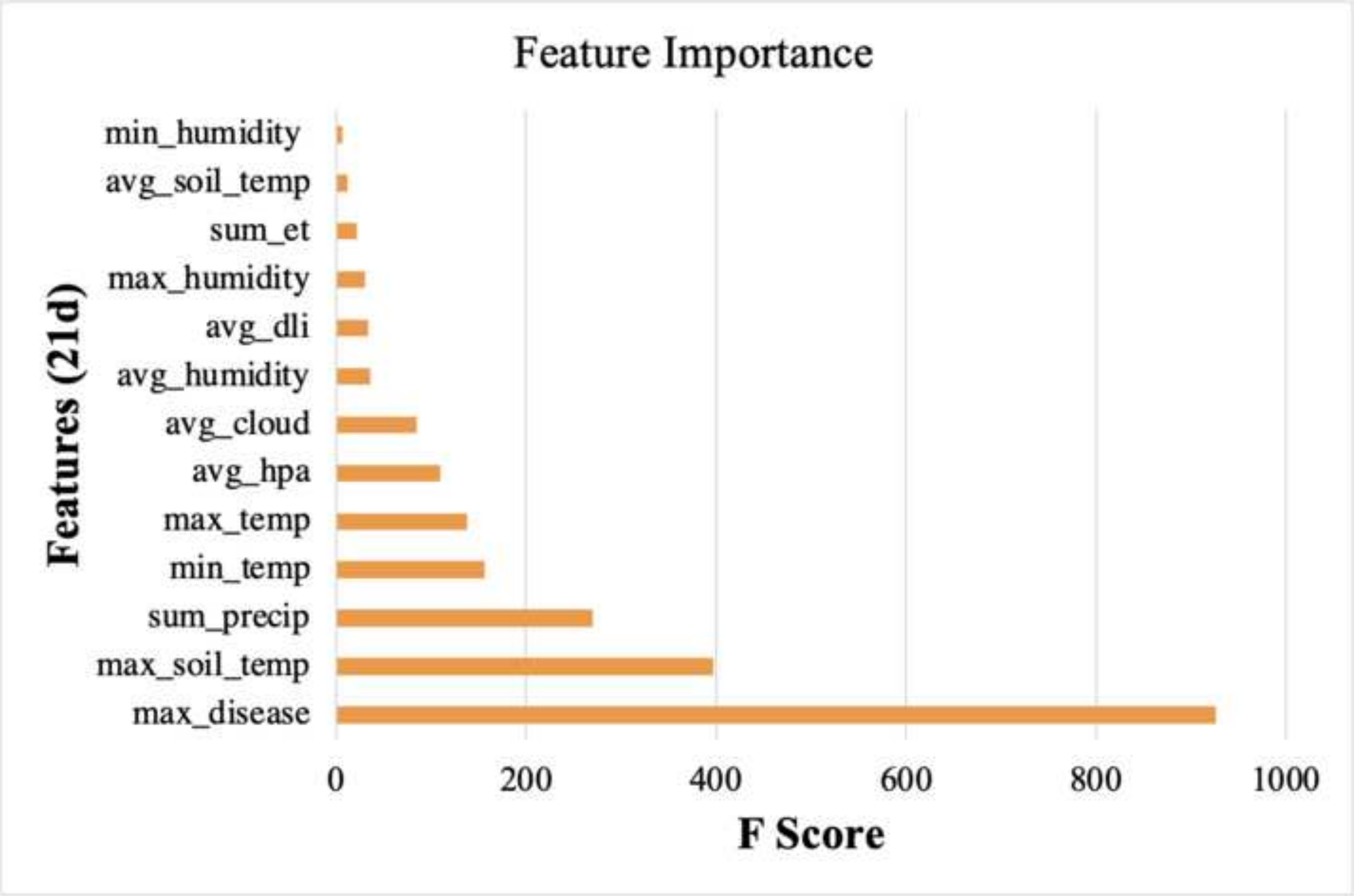

Figure 5

Click here to access/download;Figure;Fig5.tiff

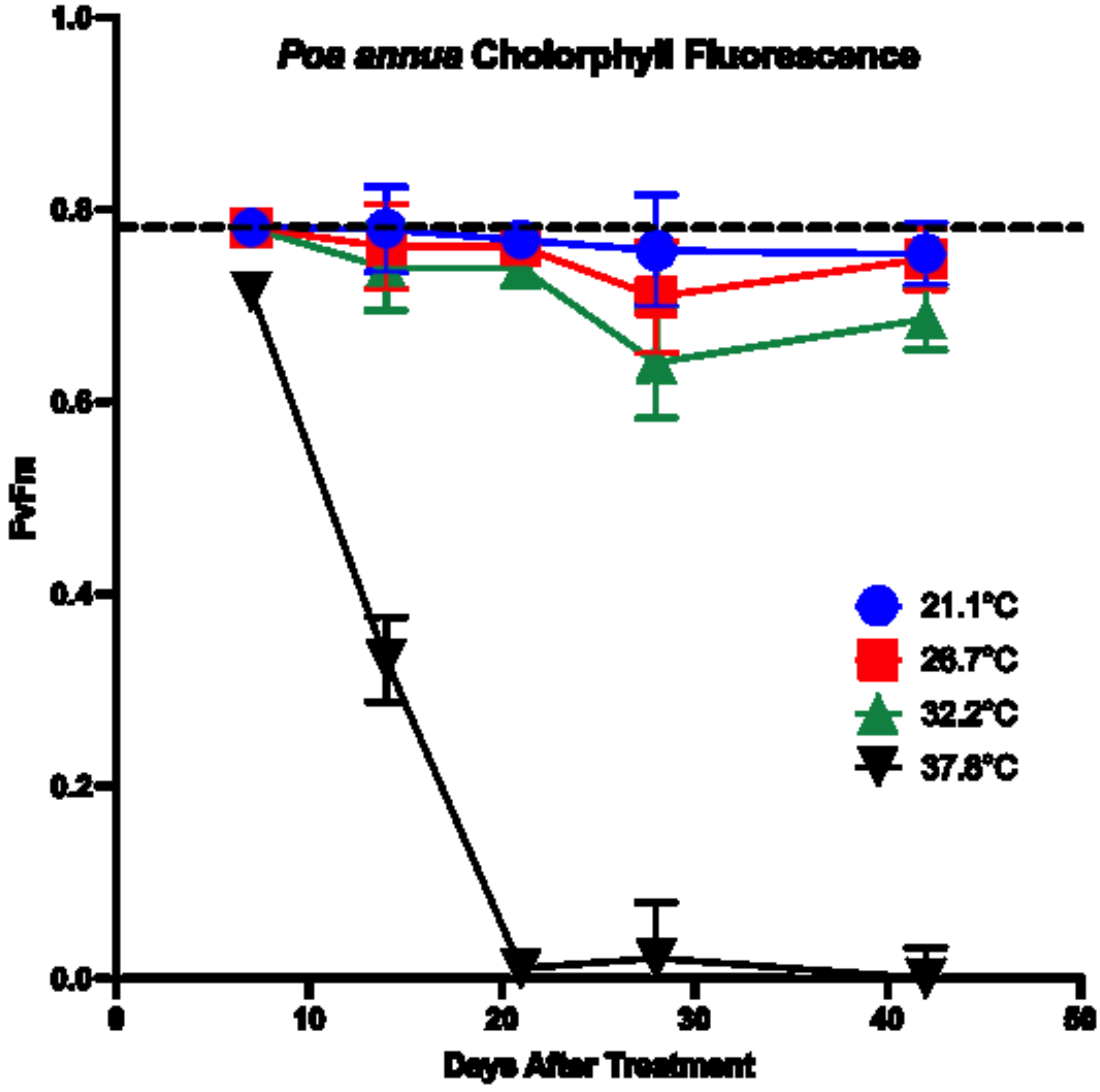

Figure 6

Click here to access/download;Figure;Fig6.tiff

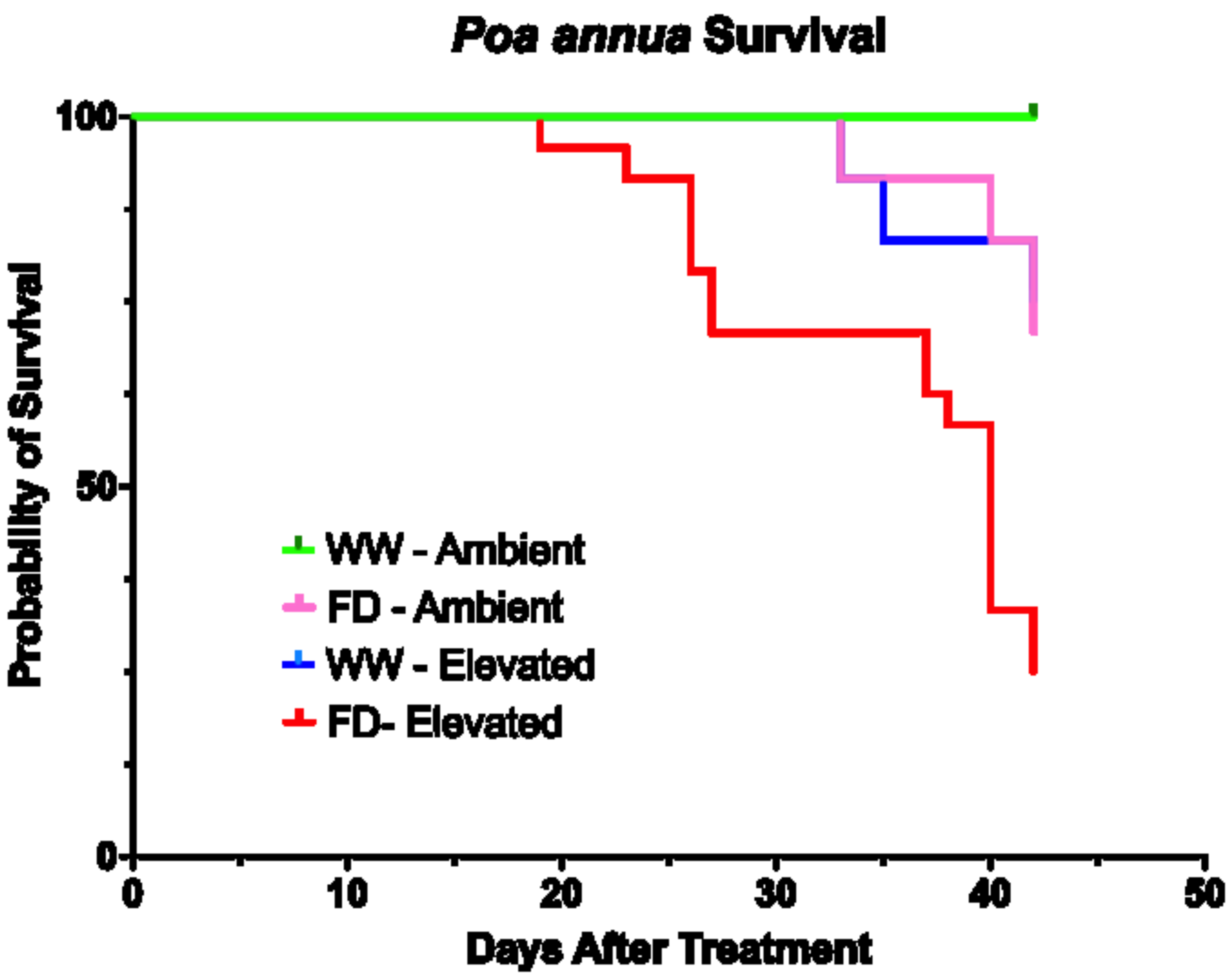

Figure 7

Click here to access/download;Figure;Fig7.tiff

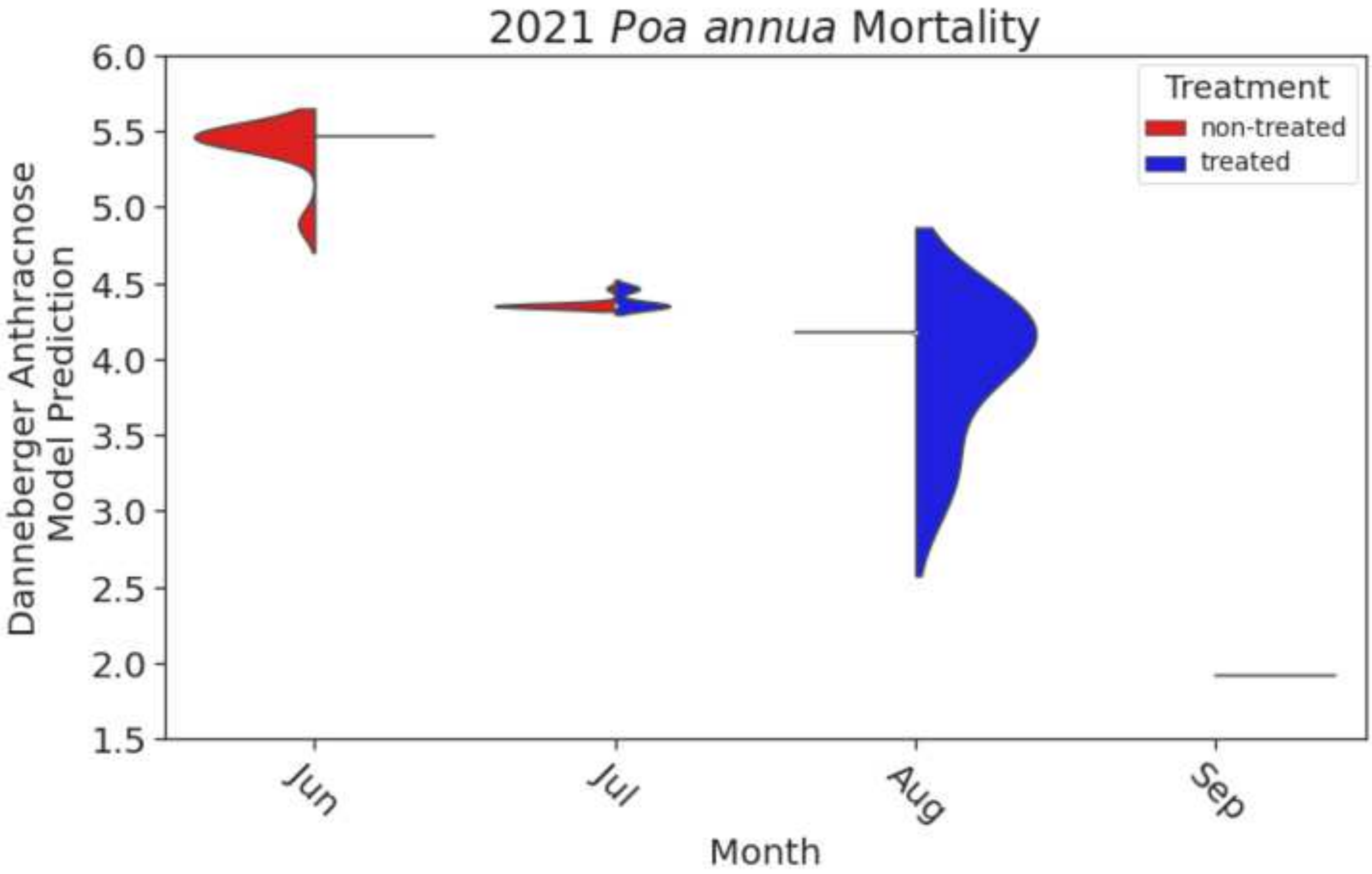

Figure 8

Click here to access/download;Figure;Fig8.tiff

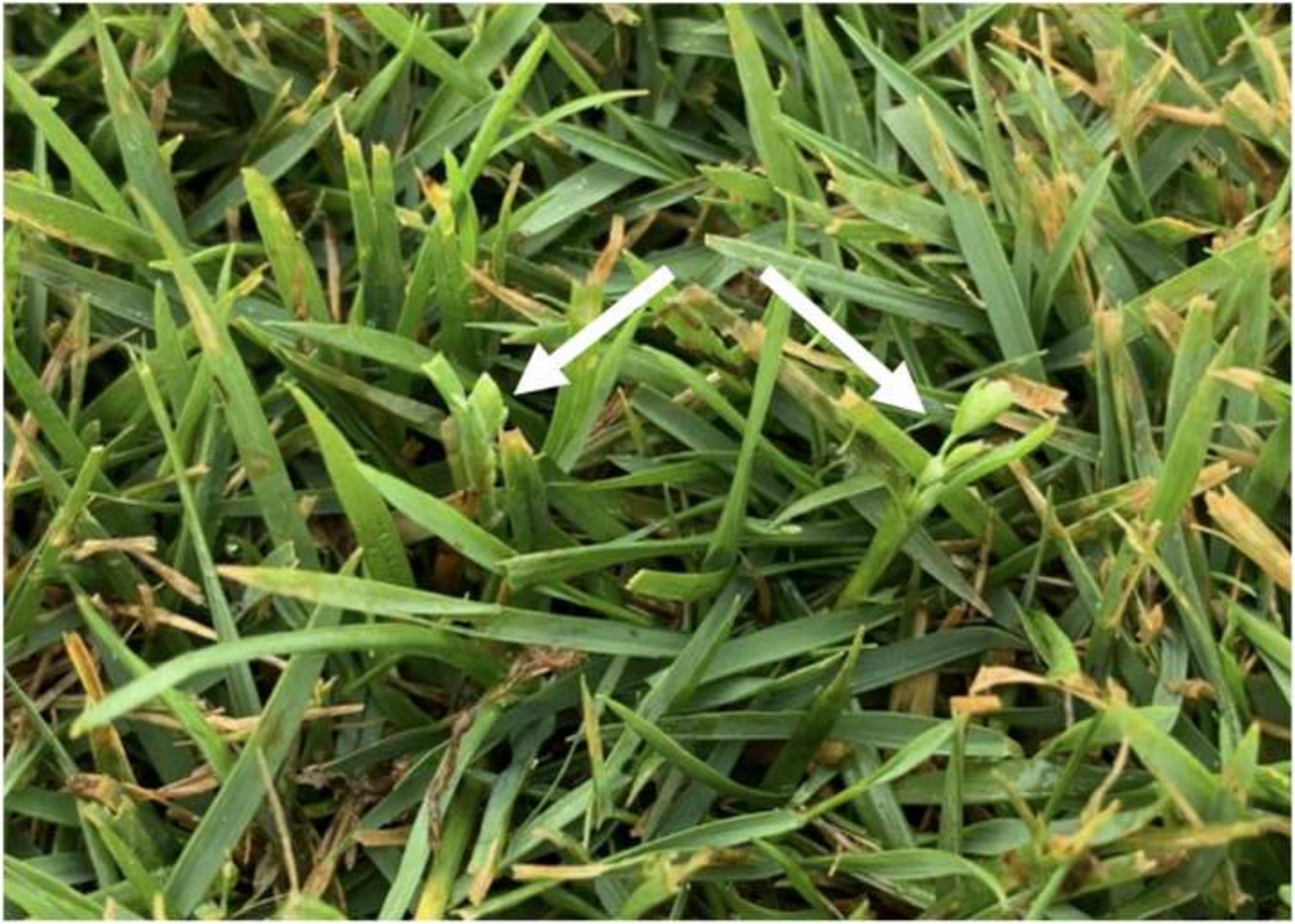

Figure 9

Click here to access/download;Figure;Fig9.tiff

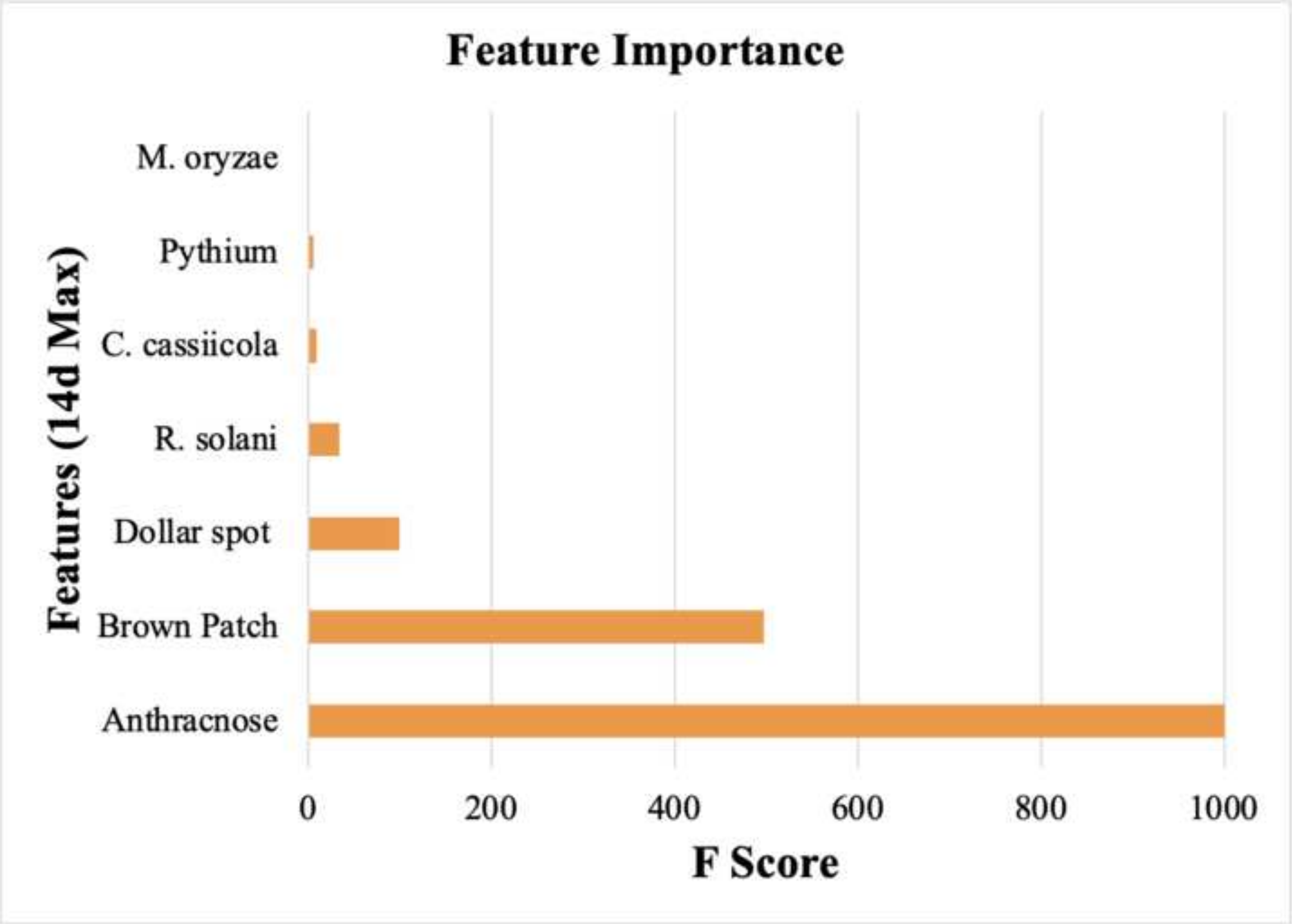

Supplement: Attachment — Submitted filename: PONE-D-22-14930_reviewer.pdf [file pone.0274404.s004.pdf]
